# Adipocyte-specific Repression of PPAR-gamma by NCoR Contributes to Scleroderma Skin Fibrosis

**DOI:** 10.1186/s13075-018-1630-z

**Published:** 2018-07-11

**Authors:** Benjamin Korman, Roberta Goncalves Marangoni, Gabriel Lord, Jerrold Olefsky, Warren Tourtellotte, John Varga

**Affiliations:** 1 Northwestern Scleroderma Program, Division of Rheumatology, Northwestern University Feinberg School of Medicine, Chicago, IL USA; 2 Division of Allergy, Immunology and Rheumatology, University of Rochester Medical Center, Rochester, NY USA; 3 Division of Endocrinology, University of California, San Diego, La Jolla, CA USA; 4 Department of Pathology, Cedars Sinai Medical Center, Los Angeles, CA USA; 5 Department of Dermatology, Northwestern University Feinberg School of Medicine, Chicago, IL USA

**Keywords:** NCoR, Adipocyte, Skin fibrosis, Scleroderma, PPAR-γ, Adipogenesis, Fibrogenesis

## Abstract

**Background:**

A pivotal role for adipose tissue homeostasis in systemic sclerosis (SSc) skin fibrosis is increasingly recognized. The nuclear receptor PPAR-γ is the master regulator of adipogenesis. Peroxisome proliferator activated receptor-γ (PPAR-γ) has antifibrotic effects by blocking transforming growth factor-β (TGF-β) and is dysregulated in SSc. To unravel the impact of dysregulated PPAR-γ in SSc, we focused on nuclear corepressor (NCoR), which negatively regulates PPAR-γ activity and suppresses adipogenesis.

**Methods:**

An NCoR-regulated gene signature was measured in the SSc skin transcriptome. Experimental skin fibrosis was examined in mice with adipocyte-specific NCoR ablation.

**Results:**

SSc skin biopsies demonstrated deregulated NCoR signaling. A 43-gene NCoR gene signature showed strong positive correlation with PPAR-γ signaling (*R* = 0.919, *p* < 0.0001), whereas negative correlations with TGF-β signaling (*R* = − 0.796, *p* < 0.0001) and the modified Rodnan skin score (*R* = − 0.49, *p* = 0.004) were found. Mice with adipocyte-specific NCoR ablation demonstrated significant protection from experimental skin fibrosis and inflammation. The protective effects were mediated primarily through endogenous PPAR-γ.

**Conclusions:**

Our results implicate, for the first time, to our knowledge, deregulated NCoR/PPAR-γ pathways in SSc, and they support a role of adipocyte modulation of skin fibrosis. Pharmacologic restoration of NCoR/PPAR-γ signaling may represent a novel strategy to control skin fibrosis in SSc.

## Background

Systemic sclerosis (SSc) is a poorly understood multisystem disorder characterized by autoimmunity, vasculopathy, and fibrosis [1, 2]. A distinguishing hallmark of SSc is simultaneous fibrosis in multiple organs [3]. SSc has high mortality and no effective therapy. Myofibroblasts originating from distinct tissue-resident progenitors are key drivers of organ fibrosis in SSc and other fibrotic conditions [4]. Recent studies have demonstrated that aberrant adipose tissue homeostasis and dysregulated adipogenesis play vital roles in SSc by contributing to myofibroblast accumulation in the fibrotic dermis [5]. Targeting the cellular transition from adipocyte to myofibroblast therefore represents a potential therapeutic approach to reduce fibrosis.

Peroxisome proliferator-activated receptor-γ (PPAR-γ) is a pleiotropic nuclear hormone receptor that is the master regulator of adipogenesis and is required for adipocyte differentiation [6]. Intriguingly, recent studies have shown that PPAR-γ has an important antifibrotic role in SSc and other fibrosing disorders [7]. PPAR-γ counteracts profibrotic TGF-β signaling [8, 9] and has been shown to have antifibrotic activities in several organs relevant to SSc, including skin, lung, and heart [10–12]. Importantly, SSc skin biopsies and explanted fibroblasts demonstrate reduced PPAR-γ expression and activity [5, 11, 13, 14]). In mice, ablation of PPAR-γ in fibroblasts promotes cutaneous fibrogenesis [15]. Moreover, genetic studies demonstrate significant associations of PPAR-γ variants with SSc, and transcriptomic studies have implicated the PPAR-γ pathway as fundamentally dysregulated in SSc skin biopsies [16–18]. Together, these findings provide strong support for the role of PPAR-γ deregulation in the pathogenesis of SSc, and they suggest that targeting PPAR-γ signaling could represent a rational therapeutic approach in fibrosis. Indeed, PPAR-γ agonists, including rosiglitazone and a novel pan-PPAR agonist IVA337, have been shown to have antifibrotic effects in mice [13, 19]. However, because PPAR-γ agonists have off-target and ligand-independent effects [6, 20–24], approaches designed to selectively target the PPAR-γ pathway may provide an alternative way to modulate fibrogenesis in SSc without the adverse side effects of PPAR-γ agonists.

The expression of PPAR-γ is tightly regulated by nuclear coreceptors (NCoRs) [25]. In the absence of ligand, PPAR-γ is complexed to retinoid X receptors (RXRs) and two corepressors, NCoR and SMRT (silencing mediator of retinoid and thyroid receptors), which prevent DNA binding and transcriptional activity [26]. Upon ligation, corepressors are displaced from the PPAR-γ/RXR complex and coactivators are recruited, allowing sequence-specific binding to conserved PPAR-γ response elements in target gene promoters [27]. NCoR is a ubiquitously expressed nuclear corepressor with potent tissue-specific effects on lipid metabolism and mitochondrial energy homeostasis [27]. In adipose tissue, the dominant function of NCoR is inhibition of PPAR-γ activity, and mice with adipocyte-specific NCoR knockout show unrestrained PPAR-γ activity [28].

In light of the fundamental role of PPAR-γ and its regulation of adipogenesis in SSc, we sought to investigate the contribution of NCoR as a key regulator of PPAR-γ in SSc skin fibrosis. We found that an NCoR-regulated gene signature is aberrantly expressed in SSc skin biopsies and correlates with PPAR-γ and TGF-β signaling as well as extent of skin disease. Mice with adipocyte-specific NCoR ablation showed amelioration of skin fibrosis that was mediated via endogenous PPAR-γ. Together, these results support a vital role for the NCoR/PPAR-γ pathways in SSc skin fibrosis. Furthermore, these findings also implicate adipocytes as a key cell type in modulating fibrosis and indicate that restoration of adipose NCoR/PPAR-γ signaling could serve as a novel approach for treating skin fibrosis.

## Methods

### Measurement of NCoR pathway score in SSc skin biopsies

To assess NCoR pathway activation in SSc, we defined an NCoR-responsive gene signature comprised of 101 genes previously shown to be specifically repressed by NCoR [29]. The expression of this signature was then queried in a microarray dataset [GEO:GSE76886] of SSc (*n* = 70) and healthy control (*n* = 22) skin biopsies. All biopsies were obtained prior to starting immunomodulatory therapy. There were 43 transcripts out of 101, with a coefficient of variation > 0.5 and a false discovery rate (FDR) < 0.05, and these transcripts were included in subsequent analyses. An NCoR pathway score was calculated on the basis of previously described approaches, and scores were normalized to the mean of the controls [30]. The standardized gene expression levels were summed for each biopsy to provide an NCoR signature score based on the following formula:${\sum}_{i=1}^n=\frac{GENEi_{SSc}-{MEANi}_{ctr}}{SDctr}\ast k$,

where *i* = each NCoR-regulated gene, Gene *i*_SSc_ = gene expression level in each SSc biopsy, and Mean *i*_ctr_ = the average gene expression in controls. For NCoR-induced genes, *k* = 1; for NCoR-suppressed genes, *k* = − 1.

In subsequent analyses, the NCoR pathway score in each skin biopsy was correlated with previously defined PPAR-γ and TGF-β gene signatures [5, 31]. In SSc biopsies with sufficient clinical data (*n* = 36), NCoR pathway scores were correlated with the modified Rodnan skin score (MRSS). Because expression data were collected from publicly available sources, detailed clinical information was available for 36 of the patients with SSc and 11 control individuals. The age of patients was 48.0 ± 11.6 (mean ± SD) years, as compared with control individuals, who were aged 39.9 ± 11.8 (mean ± SD) years. Among patients with SSc, 86.1% female, compared with 63.6% of control individuals. Among patients with SSc, 30 had diffuse cutaneous disease (83.3%), 5 had limited cutaneous disease, and 1 had overlap SSc, and the MRSS was 17 ± 10 (mean ± SD). Twelve patients (33%) had early disease (< 2 years disease duration since onset of first non-Raynaud’s symptom), and 24 (67%) had late disease (> 2 years disease duration since onset of first non-Raynaud’s symptom).

#### Animals

Mice carrying floxed alleles of NCoR on a C57BL/6J background were backcrossed to mice harboring Cre recombinase driven by the adipocyte protein 2 promoter to create NCoR^flx/flx^ (wild-type [WT]) and NCoR^flx/flx^-ap2Cre (adipocyte nuclear corepressor-knockout [AKO]) animals [28]. Genotypes of all animals were confirmed. All experimental procedures complied with the Public Health Service Policy on Humane Care and Use of Laboratory Animals, and protocols were approved by the institutional animal care and use committee of Northwestern University.

#### Diets

Mice were fed a standard chow diet (LM-485, 17% kcal from fat; Harlan Teklad, Madison, WI, USA) until 8–12 weeks of age and were subsequently switched to a high-fat diet containing 60% fat (D12492; Research Diets Inc., New Brunswick, NJ, USA).

#### Bleomycin-induced fibrosis

Mice were treated with bleomycin (1 mg/ml in PBS; 10 mg/kg/d) or PBS by daily subcutaneous injection for 14 days and killed at 21 days [32, 33]. In some experiments, mice received concurrent daily intraperitoneal injections of the PPAR-γ inhibitor GW9662 (Sigma-Aldrich, St. Louis, MO, USA) (1 mg/kg) for 21 days. At the time mice were killed, skin, gonadal and perirenal fat, and 16-hour fasting serum samples were harvested and processed for analysis. Each experimental group consisted of three to five mice per group, and experiments were repeated two or three times with consistent results. In other experiments, old (> 40 weeks) and young adult (< 20 weeks) mice and male and female mice were used.

### RNA isolation and qRT-PCR

Upon harvest, tissues (skin, adipose, lung, liver) were immersed in Ambion RNA*later* (Life Technologies, Carlsbad, CA, USA) and stored at − 80 °C. The samples were homogenized; RNA was isolated using the RNeasy Micro Kit or RNeasy Fibrous Mini Kit (Qiagen, Valencia, CA, USA); and quantification of gene expression was performed as previously described [34].

#### Serum assays

Fasting serum was assayed for glucose by spectrophotometry (K606-100; BioVision Inc., Milpitas, CA, USA) and for insulin by ELISA (EZRMI-13K; MilliporeSigma, Burlington, MA, USA). Homeostatic model assessment of insulin resistance (HOMA-IR) was calculated using the formula HOMA-IR = glucose × insulin/400. Fasting serum levels of leptin, adiponectin, resistin, and plasminogen activator inhibitor-1 (PAI-1) were determined by multiplex Luminex assays according to the manufacturer’s instructions (MADKMAG-71K and MADPNMAG-70K-01; MilliporeSigma).

#### Adipocyte size and numbers

The number and size of the adipocytes in the perirenal and intradermal adipose depots were evaluated in H&E-stained sections at 100 × magnification [28]. Adipocyte diameters (*n* = 250 [perirenal] and *n* = 100 [intradermal]) were determined in three noncontiguous high-power fields (HPFs) per mouse from three or four mice per group using Fiji software [35], and data were used to generate frequency distribution histograms of adipocyte diameters.

#### Histopathological analysis

Tissues were fixed in 4% phosphate-buffered paraformaldehyde (pH 7.4) and embedded in paraffin, and 4-μm-thick sections were stained with H&E. Dermal thickness (distance from the epidermis-dermis junction to the dermis-fat junction) and intradermal adipose layer thickness (distance from the dermis-fat junction to the fat-muscle junction) were determined at five randomly selected regions per HPF using Fiji software [32, 34]. To evaluate collagen deposition and organization in the dermis, sections were stained with Masson’s trichrome and Picrosirius red, and intensity was measured from two HPFs per mouse and two mice per group using Fiji software.

#### IHC

Paraffin-embedded sections of lesional skin were incubated with rabbit anti-NCoR (1:100, PA1-844A; Thermo Fisher Scientific, Waltham, MA, USA) rabbit anti-α-smooth muscle actin (α-SMA, 1:200; Sigma-Aldrich), rat anti-F4/80 (1:1000; eBioscience, San Diego, CA, USA), and rat anti-phosho-SMAD2 (1:200; Sigma-Aldrich) primary antibodies [36]. Secondary mouse antibodies were used to localize primary antibody binding. Negative controls without primary antibody were used to confirm specificity. Staining intensity of F4/80-immunopositive cells was determined from two randomly chosen regions of the dermis and the intradermal adipose layer using Fiji software.

#### Determination of collagen content

Lesional skin was hydrolyzed in HCl, and hydroxyproline content was determined using a colorimetric assay kit (BioVision, Inc.).

#### Statistical analysis

Data are presented as mean ± SEM. Statistical analyses were performed using Prism 6 software (GraphPad Software, La Jolla, CA, USA). For in vivo studies, statistical significance was assessed by Wilcoxon-Mann-Whitney test for two-group comparisons, and analysis of variance was used for multiple group comparisons. *p* < 0.05 was considered statistically significant.

## Results

### Deregulated NCoR signaling in SSc skin biopsies

#### NCoR-regulated genes

**Fig. 1:**
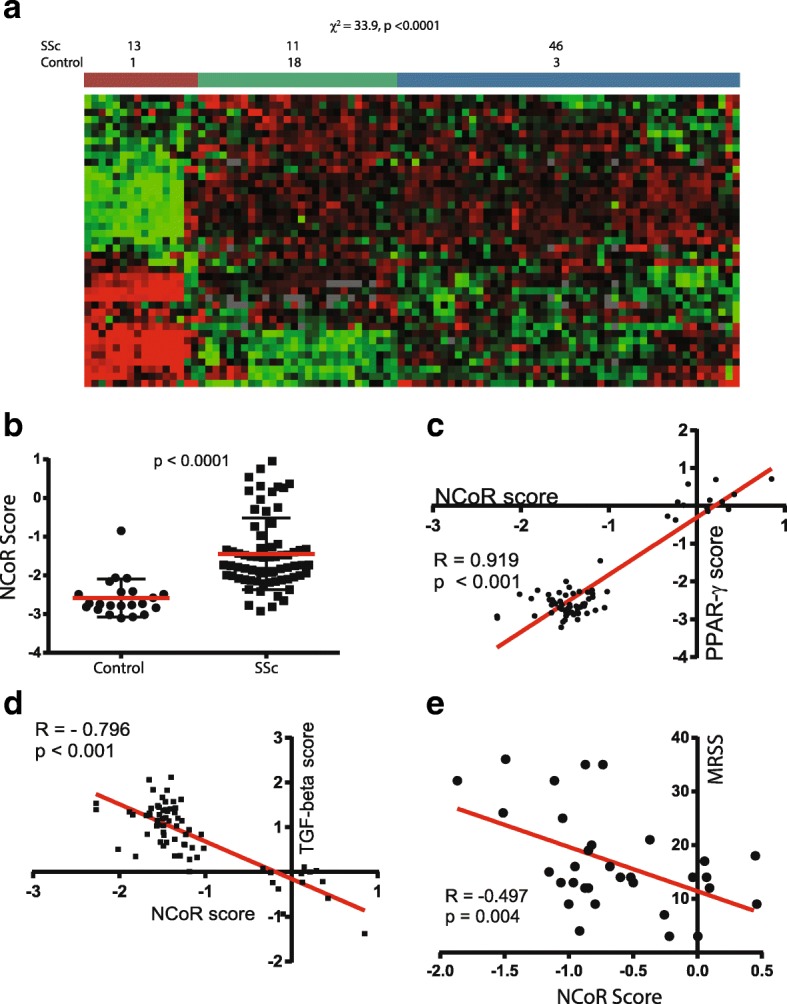
Differential expression of nuclear corepressor (NCoR)-modulated genes in systemic sclerosis (SSc). **a** Skin biopsy transcriptomes from 70 patients with SSc and 22 healthy control subjects [GEO:GSE76886] were queried for expression of genes negatively regulated by NCoR. Hierarchical clustering identified three subsets: cluster 1, a small subset with significant NCoR upregulation and comprised mostly of patients with SSc; cluster 2, a subset made mostly of control individuals; and cluster 3, a group of patients with SSc with decreased NCoR-mediated expression. These clusters distinguish SSc biopsies from controls (*p* < 0.0001 by chi-square test). The number and percentage of biopsies are denoted for each group. *Red* indicates gene upregulation; *green* indicates downregulation. **b** NCoR pathway scores were determined (*see* the Methods section for derivation). Note the significantly elevated scores in SSc skin biopsies. **c** and **d** NCoR pathway scores correlate significantly with peroxisome proliferator activated receptor-γ (PPAR-γ) and transforming growth factor-β (TGF-β) pathway scores. **e** Correlation of NCoR pathway scores and the modified Rodnan skin score (MRSS)

We first sought to examine the pattern of genes whose expression was modulated by the PPAR-γ corepressor NCoR in SSc skin biopsies. Forty-three NCoR-specific transcripts (defined in the Methods section above and referred to in this paper as the *NCoR gene signature*) were found to be differentially regulated (FDR < 0.05) in SSc skin biopsies relative to controls. Hierarchical clustering of biopsies based on this NCoR gene signature identified three clusters that robustly discriminated SSc from control biopsies (chi-square = 33.9, *p* < 0.0001 for cluster identity) (Fig. 1a). Among patients with SSc, no differences across the three clusters were found in age, sex, disease duration, or MRSS.

In an orthogonal approach, we identified a 45-gene lipid metabolism gene module (defined using KEGG pathway terms) selected from 400 genes coexpressed with NCoR (Spearman’s *r* > 0.5), and we used this module to query skin transcriptomes [GEO:GSE76886]. This set of genes used for classification identified two groupings that significantly discriminated SSc from control skin biopsies (chi-square for group identity = 10.5, *p* < 0.0001). NCoR score did not differ in either the limited vs diffuse SSc groups (*p* = 0.48) or the early vs late disease groups (*p* = 0.16). On the basis of these observations using complementary analytic approaches, we conclude that deregulated NCoR signaling is a hallmark of SSc skin.

#### NCoR signaling is correlated with PPAR-γ and TGF-β signaling

We found that NCoR pathway scores were significantly elevated in SSc biopsies compared with controls (Fig. 1b). Importantly, the NCoR pathway score showed strong positive correlation with a previously defined PPAR-γ gene signature (*R* = 0.919, *p* < 0.0001) (Fig. 1c). In sharp contrast, the NCoR pathway score was negatively correlated with a TGF-β gene signature score defined using two complementary approaches. The first TGF-β signature was defined on the basis of a clinical trial with genes showing significant change in expression in skin biopsies from patients with SSc treated with anti-TGF-β monoclonal antibody (*R* = − 0.796,*p* < 0.0001) (Fig. 1d) [37]. An alternative TGF-β-regulated gene signature derived from normal and SSc skin fibroblasts treated with TGF-β showed a similar significant correlation with the NCoR pathway (*R* = − 0.594, *p* = 0.02) (data not shown) [31]. These findings indicate that NCoR-regulated gene expression positively correlated with antifibrotic PPAR-γ signaling and negatively correlated with fibrotic TGF-β signaling in the skin of patients with SSc.

#### NCoR signaling is associated with extent of SSc skin disease

To pursue the clinical relevance of these changes, we examined their association with MRSS, a validated measure of global skin involvement. Although local MRSS may be a better predictor of local gene expression, total-body MRSS correlates closely with forearm MRSS and has been used widely as a surrogate for disease severity [38–42]. As shown in Fig. 1e, NCoR pathway scores were negatively correlated with MRSS (*R* = − 0.49, *p* = 0.004). The alternate NCoR pathway score (defined using the 45-gene NCoR coexpressed lipid metabolism module) demonstrated similar correlation with MRSS (*R* = 0.46, *p* = 0.01). Together, these orthogonal approaches indicate that NCoR-modulated gene expression is biologically relevant to pathways fundamental to fibrosis in SSc and has clinically significant associations. These findings prompted us to seek experimental evidence to define the role of NCoR in skin fibrosis.

### Mice with NCoR adipocyte-specific knockout show PPAR-γ activation

**Fig. 2:**
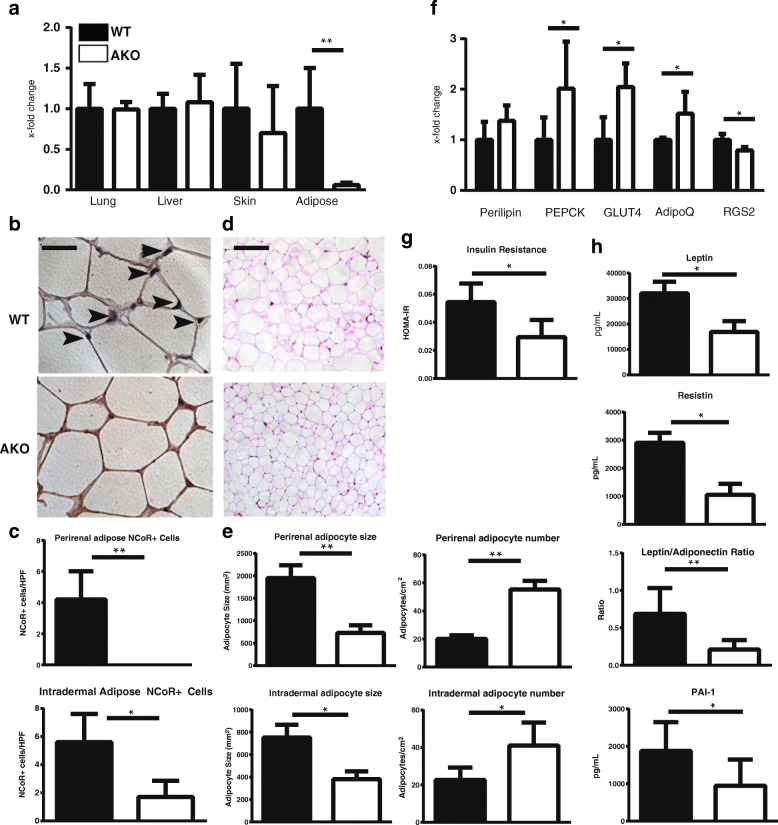
Characterization of adipocyte nuclear corepressor-knockout (ap2-NCoR^flx/flx^; AKO) mice. Skin, serum, lung, liver, and adipose tissues were harvested from untreated AKO and littermate control mice on a high-fat diet. Tissues were assessed for histologic and biochemical changes suggestive of alterations in adipocyte function. **a** Nuclear corepressor (NCoR) expression is significantly reduced in adipose tissue but not in lung, liver, or skin. Tissue was harvested from 3- to 4-week-old mice, and messenger RNA (mRNA) levels were determined by qPCR. The results represent fold changes in triplicate determinations from three or four mice per group and normalized to GAPDH. ** *p* < 0.01 by Mann-Whitney *U* test. *Black bars*, wild-type (WT) mice; *white bars*, AKO mice. **b** NCoR IHC staining of perirenal adipose tissue evident in WT mice was not detectible in AKO mice. *Arrowheads* indicate nuclei with positive staining for NCoR. Representative images are shown. Scale bars = 100 μm. **c** Quantification of NCoR+ cells in perirenal and intradermal adipose tissue (cells per high-power field [HPF]). The results are derived from 5 HPFs from two or three mice per group. * *p* < 0.05 by Mann-Whitney *U* test. **d** AKO mice have reduced expansion of adipocyte size and increased adipocyte number. Representative images of H&E staining of perirenal adipose tissue from mice fed a high-fat diet. Scale bars = 100 μm. **e** Quantification of adipocyte size (left) and number (right) in perirenal (top) and intradermal (bottom) adipocytes. Adipocyte diameter and number (area results derived from > 200 adipocytes/mouse and number quantified from 2 to 4 HPF/mouse from three or four mice per treatment group). Data presented are mean ± SD. * *p* < 0.05 by Mann-Whitney *U* test. **f** Ablation of NCoR is associated with increased expression of PPAR-γ-responsive genes. Gonadal adipose tissue was harvested, and mRNA levels were analyzed by qRT-PCR. The results represent fold changes compared with littermate controls. Data were obtained in triplicate determinations from three or four mice per group and normalized to GAPDH. * *p* < 0.05 by Mann-Whitney *U* test. **g** AKO mice have improved insulin sensitivity. The homeostatic model assessment of insulin resistance (HOMA-IR) was calculated from fasting serum insulin and glucose measurements in mice fed high fat diet for 15 weeks, * *p* < 0.05 by Mann-Whitney *U* test. **h** AKO mice have altered circulating adipokine levels. Adipokines were measured in serum of fasting mice using multiplex Luminex assays (*n* = 4–5 mice/group; * *p* < 0.05 by Mann-Whitney *U* test). *PEPCK* Phosphoenolpyruvate carboxykinase, *GLUT4* Glucose transporter type 4, *RGS2* Regulator of G-protein signaling 2, *PAI-1* Plasminogen activator inhibitor -1

In order to investigate the potential role of NCoR in the development of fibrosis, we took advantage of a mouse model with adipocyte-specific ablation of NCoR: the ap2-Cre-NCoR^*flx/flx*^ (AKO) mouse [28]. On a high-fat diet, AKO mice demonstrated > 90% lower NCoR levels in adipose tissue than control mice; most other tissues, including lung and liver, showed no significant decrease (Fig. 2a). Skin from AKO mice demonstrated a 30% decrease in NCoR expression, reflecting the loss of intradermal fat. Adipose tissue-specific loss of NCoR in AKO mice was confirmed by IHC of perirenal (visceral) and intradermal white adipose depots (Fig. 2b, c). The size of adipocytes in these fat depots was markedly decreased (*p* = 0.0006), whereas the number of adipocytes was increased (*p* = 0.008), in AKO mice compared with littermate controls, consistent with increased adipogenesis in both visceral and intradermal adipose tissues (Fig. 2d and e) [43]. Skin biopsies from AKO mice demonstrated upregulation of PPAR-γ target genes perilipin, PEPCK, GLUT4, and adiponectin and downregulation of RGS2, a negative regulatory target, in the adipose layer (Fig. 2f). Furthermore, AKO mice showed improved insulin sensitivity (HOMA-IR), whereas circulating levels of the adipokines leptin, resistin, and PAI-1 were reduced in the serum (Fig. 2g). The ratio of leptin/adiponectin, considered a marker of healthy adipose tissue [44], was reduced in serum from AKO mice (Fig. 2h). Together, these findings indicate that ablation of NCoR in adipose tissue is associated with de-repression of PPAR-γ and enhanced cell-autonomous PPAR-γ activity.

### NCoR AKO mice are resistant to skin fibrosis

**Fig. 3:**
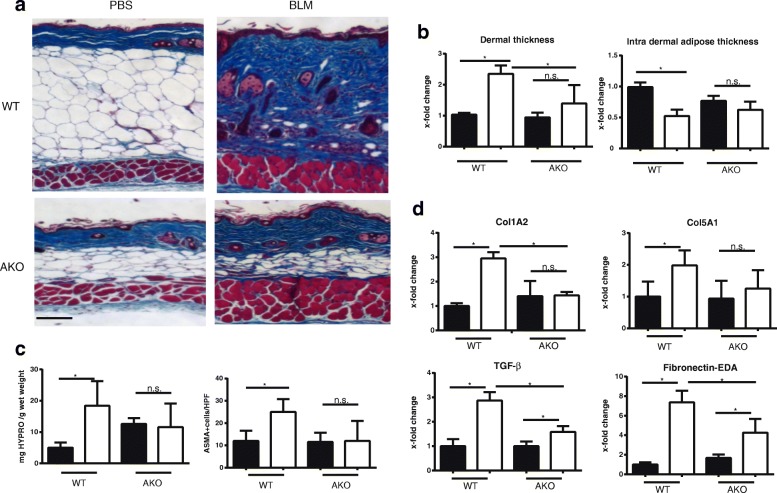
Adipocyte nuclear corepressor-knockout (AKO) mice are protected from skin fibrosis. Twenty-eight- to 32-week-old AKO mice and littermate controls (wild type [WT]) on a high-fat diet for 20 weeks were given daily subcutaneous injections of bleomycin (BLM) or PBS for 14 days, and skin was harvested at 21 days. **a** Increased dermal collagen deposition and loss of intradermal fat are attenuated in AKO mice. Masson’s trichrome stain. Representative images. Scale bars = 100 μm. **b** Quantification of dermal and intradermal adipose layer thickness. Assessments (fold change relative to controls) were performed in four or five mice per group and in five high-power fields per mouse. Data presented are mean ± SD, * *p* ≤ 0.05 by analysis of variance (ANOVA). **c** Reduced collagen accumulation. Left, hydroxyproline (HYPRO) assays, adjusted for wet weight; right, IHC for α-smooth muscle actin (ASMA). ASMA+ cells in the dermis were counted by a blinded observer in three randomly selected high-power fields in three mice per group. Results represent mean ± SEM. * *p* < 0.05 by ANOVA. **d** Fibrotic gene expression in PBS-treated (*black bars*) or BLM-treated (*white bars*) mice. Messenger RNA levels were determined by qRT-PCR. Results represent fold changes compared with control in triplicate determinations from three to five mice per group and normalized to *GAPDH*. * *p* < 0.05 by Mann-Whitney *U* test. *EDA* Extra domain A, *TGF-β* Transforming growth factor-β, *Col1A2* Collagen type I, α2 chain, *Col5A1* Collagen type V, α1 chain

To evaluate the impact of adipocyte-specific NCoR loss on fibrogenesis, we used the well-characterized bleomycin model [45]. AKO mice and littermate controls maintained on a high-fat diet were challenged with subcutaneous bleomycin or PBS. Under high-fat diet conditions, control mice showed a dramatic enhancement of the intradermal adipose tissue, with increased adipocyte size and number, as expected (Figs. 2d and 3a). In contrast, AKO mice were almost completely resistant to high-fat diet-induced intradermal adipocyte hypertrophy. At day 21 of bleomycin treatment, WT mice showed a significant increase in dermal thickness (*p* < 0.001) and collagen deposition, as well as attenuation of the intradermal adipose layer (*p* = 0.02), compared with vehicle-treated mice (Fig. 3a). Identically treated NCoR-deficient mice showed significantly attenuated increases in dermal thickening (*p* = 0.02) and intradermal adipocyte loss (*p* = 0.04) (Fig. 3b). Consistent results were obtained in three independent experiments, demonstrating reproducible amelioration of dermal fibrosis and maintenance of intradermal adipose tissue in both young (< 20 weeks old) and old (> 40 weeks old) mice, as well as in male and female mice (data not shown) lacking adipocyte NCoR. The extent of dermal collagen deposition and myofibroblast numbers were both reduced in AKO mice (Fig. 3c). Further analysis of the lesional skin demonstrated significant reduction in the expression of numerous fibrotic genes (Col1A2, Col 5A1, fibronectin-EDA, and TGF-β) (Fig. 3d). Together, these results indicate that loss of NCoR in adipocytes is sufficient to protect mice against experimentally induced skin fibrosis.

### Pharmacologic inhibition of PPAR-γ reverses the antifibrotic phenotype of AKO mice

**Fig. 4:**
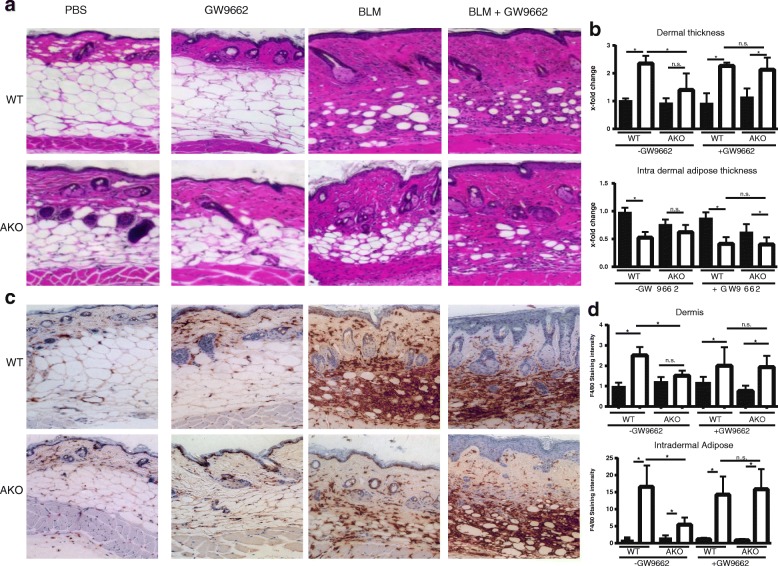
Protection from fibrosis in adipocyte nuclear corepressor-knockout (AKO) mice is mediated by peroxisome proliferator activated receptor-γ (PPAR-γ). Twenty- to 30-week-old AKO and wild-type (WT) littermate control mice were administered PBS or bleomycin (BLM) for 14 days, and lesional skin was harvested for analysis. The selective PPAR-γ inhibitor GW9662 (Sigma-Aldrich) was coadministered by intraperitoneal injection for 21 days. Injections were given as previously described (Refs [19] and [45]) as daily is not quite accurate. **a** Inhibition of PPAR-γ reversed the nuclear corepressor’s antifibrotic effect. H&E stain; representative images are shown. Scale bars = 100 μm. **b** Quantification of dermal (top) and intradermal adipose layer thickness (bottom). Results represent fold changes relative to control mice in two three mice per treatment condition and five high-power field areas per mouse; mean ± SD. * *p* ≤ 0.05 by analysis of variance. Results were consistent across two different experiments. **c** IHC was performed for macrophages; F4/80 staining is shown. **d** Relative F4/80 staining intensity in the dermis and intradermal adipose tissue was determined in two randomly chosen regions using Fiji software. Results represent mean ± SEM. * *p* < 0.05

In view of the pleiotropic and cell-type-specific functions of NCoR regulating multiple signaling pathways [27], we sought to identify whether the antifibrotic effects observed in mice lacking adipocyte NCoR were mediated through PPAR-γ, a primary target regulated by NCoR. For this purpose, we used the irreversible PPAR-γ antagonist 2-chloro-5-nitrobenzanilide (GW9662) [46]. GW9662 was administered by intraperitoneal injection (1 mg/kg/d) concurrently with bleomycin or vehicle. In AKO mice, GW9662 largely abrogated protection from bleomycin-induced fibrosis, whereas on its own GW9662 had no effect. GW9662 on its own had no effect on skin fibrosis in WT mice (Fig. 4a). Moreover, bleomycin-treated AKO mice demonstrated a reduction in F4/80-positive macrophage accumulation compared with similarly treated WT mice, which was reversed by cotreatment with GW9662 (Fig. 4b). We conclude that the fibrotic impact of adipocyte NCoR involves repression of PPAR-γ and that protection from fibrosis seen in NCoR-deficient mice is mediated via antifibrotic activities of endogenous PPAR-γ.

## Discussion

In this study, we investigated the effect of adipocyte-specific loss of the PPAR-γ corepressor NCoR on skin fibrosis and its expression in patients with SSc. The results indicate that adipose-specific NCoR ablation ameliorates the development of dermal fibrosis in mice. Moreover, skin fibrosis in SSc is accompanied by increased NCoR activity and decreased expression of PPAR-γ-regulated genes. The antifibrotic effect of NCoR ablation involves cell-autonomous increased PPAR-γ signaling, blockade of TGF-β-dependent fibroblast activation, and attenuation of the cutaneous inflammatory response. Furthermore, loss of NCoR in adipocytes counteracts the depletion of dermal adipose tissue via augmented PPAR-γ activity. Together, these findings indicate that loss of NCoR repression and consequent PPAR-γ activation in adipocytes protect against skin fibrosis. These findings are consistent with the notion that PPAR-γ plays an endogenous antifibrotic role in the skin and suggest that therapies targeting the NCoR/PPAR- γ pathway and adipogenesis represent novel therapeutic approaches for SSc.

Analysis of skin biopsy transcriptomes suggested a link between NCoR signaling and skin fibrosis in SSc. We used two distinct approaches to ascertain NCoR signaling and found that both differentiated SSc biopsies from healthy controls and identified novel SSc subsets with aberrant NCoR signaling. When measured as a pathway score, NCoR activation demonstrated a strong positive correlation with PPAR-γ signaling in SSc skin biopsies (*R* = 0.919) and a strong negative correlation with TGF-β signaling (*R* = 0.792). These correlations are consistent with the idea that in addition to its role as a PPAR-γ corepressor, NCoR downregulates the expression of genes involved in TGF-β signaling. The correlation of NCoR pathway activity with the MRSS potentially implicates NCoR/PPAR-γ in SSc fibrosis and importantly suggests that these molecular findings have implications for the extent of clinical skin disease in SSc.

Mice deficient in adipocyte NCoR demonstrate the importance of this corepressor in dermal fibrosis. NCoR exerts diverse effects that are pleiotropic and tissue-specific. Previous work has demonstrated that NCoR plays a primary role in repressing the expression PPAR-γ in adipose tissue [28]. We ablated NCoR in adipose tissue to assess whether consequent PPAR-γ gain of function could modulate dermal fibrosis. We recently demonstrated a critical role of intradermal adipose tissue homeostasis in the pathogenesis of fibrosis and implicated dermal adipocytes as progenitors of fibrotic myofibroblasts [32]. We therefore hypothesized that de-repression of PPAR-γ could promote adipogenesis in the skin and attenuate dermal fibrosis. Indeed, we found that AKO mice treated with bleomycin showed consistent and significant reductions in dermal thickness and collagen accumulation. Consistent with prior studies that showed a role for adipocytes in skin fibrosis, we found that reduced skin fibrosis resulted from an adipocyte-specific NCoR ablation, therefore further supporting the notion that NCoR signaling in adipocytes is sufficient to limit fibrosis. These findings support the hypothesis that adipocytes play a central regulatory role in cutaneous fibrosis [32, 47].

NCoR is a transcriptional coregulatory protein that recruits histone deacetylases to DNA promoter regions and functions as a key regulator of nuclear receptors in the downregulation of gene expression [27]. This repression is relevant to multiple processes in cellular homeostasis, including control of blood sugar, muscle metabolism, and cell cycle regulation [26]. Whereas NCoR regulates many other molecules, including other PPAR family members in other tissues, and is more specific to PPAR-β and PPAR-δ in muscle, in adipose tissue NCoR is PPAR-γ-specific [28, 48, 49]. To confirm whether the antifibrotic effect of NCoR was PPAR-γ-specific in this model, we used a specific PPAR-γ inhibitor. This finding that GW9662 reversed the effects of NCoR ablation reiterates the importance of the PPAR-γ pathway in SSc, confirms that NCoR’s antifibrotic effect in SSc skin fibrosis is mediated primarily by PPAR-γ, and confirms that the PPAR-γ pathway is necessary to resisting fibrogenesis.

The exact mechanisms underlying the antifibrotic effects of NCoR remain unclear. Our data suggest that NCoR deficiency reduces macrophage accumulation in the dermis (Fig. 4c). Additionally, NCoR increases omega-3 fatty acids [50]. This effect may explain why NCoR deficiency in our AKO mice did not add additional protection from fibrosis when fed a fish oil-based high-fat diet (data not shown). Further functional studies to characterize NCoR’s molecular interactions are needed to describe how corepression and altered PPAR-γ signaling may lead to an altered antifibrotic phenotype. Moreover, whether NCoR modulates fibrosis in other organs such as the lung, where there is no adipose tissue, remains unknown and should be further investigated. Although this study demonstrates that adipose tissue is of primary importance, NCoR’s role in repressing PPAR-γ in other cell populations should also be investigated.

Despite the theoretical advantages of activating PPAR-γ directly for therapy, the ubiquitous expression and multiple off-target and ligand-independent effects of PPAR agonists have resulted in multiple adverse effects in clinical trials [6]. These include fluid retention, weight gain, bone loss, and congestive heart failure, all of which could be particularly deleterious in patients with SSc [6, 23]. In view of these concerns, targeting PPAR-γ by de-repressing its expression in adipocytes represents an attractive alternative antifibrotic strategy in SSc without causing unwanted side effects. On the basis of our results, targeting adipogenesis represents an innovative approach to control skin fibrosis in SSc. Molecules that target PPAR-γ in a ligand-independent fashion may be able to promote antifibrotic properties while avoiding some of the PPAR-γ side effects seen with current thiazolidinedione drugs. Moreover, NCoR interacts significantly with histone deacetylase 3 (HDAC3), and this study suggests that targeting the NCoR/HDAC3 complex may have beneficial and antifibrotic effects without some of the nonspecific side effects associated with pan-HDAC inhibitors [51].

## Conclusions

This work demonstrates, for the first time, to our knowledge, that the corepressor NCoR, which negatively regulates the activity of adipocyte PPAR-γ, is altered in SSc skin. Loss of NCoR corepression in adipocytes is associated PPAR-γ-mediated protection from skin fibrosis in a mouse model. Our findings highlight the important role of adipocytes in modulating SSc skin fibrosis and the vital antifibrotic role of PPAR-γ signaling. Targeting the NCoR and PPAR-γ pathways may represent a novel and rational treatment strategy for patients with SSc with alterations in these vital pathways.

## Abbreviations

AKO: Adipocyte nuclear corepressor-knockout mice
ANOVA: Analysis of variance
ASMA: α-Smooth muscle actin
BLM: Bleomycin
FDR: False discovery rate
GLUT4: Glucose transporter type 4
HOMA-IR: Homeostatic model assessment of insulin resistance
HPF: High-power field
HYPRO: Hydroxyproline
mRNA: Messenger RNA
MRSS: Modified Rodnan skin score
NCoR: Nuclear corepressor
PAI-1: Plasminogen activator inhibitor -1
PEPCK: Phosphoenolpyruvate carboxykinase
PPAR-γ: Peroxisome proliferator activated receptor-γ
RGS2: Regulator of G-*protein* signaling 2
RXR: Retinoid X receptor
SSc: Systemic sclerosis
SMRT: Silencing mediator of retinoid and thyroid receptors
TGF-β: Transforming growth factor-β
WT: Wild type
